# Maternal Knowledge and Practices on Complementary Feeding and Associated Factors in Sedal District, Western Ethiopia

**DOI:** 10.1002/fsn3.70286

**Published:** 2025-05-19

**Authors:** Habtamu Fekadu Gemede, Kassahun Ayele, Meron Demisew

**Affiliations:** ^1^ Department of Food and Nutritional Sciences Wollega University Nekemte Ethiopia

**Keywords:** complementary feeding, Ethiopia, knowledge, practices

## Abstract

Maternal knowledge and practices regarding complementary feeding are crucial determinants of children's nutrition and health. However, significant challenges remain owing to insufficient understanding and suboptimal compliance with the recommended guidelines. This study aimed to assess maternal knowledge and practices regarding complementary feeding in Sedal District, Western Ethiopia. A community‐based cross‐sectional study was conducted in Sedal District from February to July 2023. This study used the cluster and random sampling methods. Data were analyzed using SPSS version 25. Multivariable logistic regression analysis was used to identify factors associated with maternal knowledge and practices. The significance of the association was determined using the adjusted odds ratio (AOR) with a 95% confidence interval (CI) of *p* < 0.05. Overall, 46.8% and 42.7% of the mothers were knowledgeable and had appropriate practices regarding their children's complementary feeding. Women's educational level (*p* < 0.01) was significantly associated with maternal knowledge of complementary feeding. Likewise, Women's education (*p* < 0.01), household monthly average income (*p* < 0.01), and family size (*p* < 0.01) were significantly associated with mothers' children's complementary feeding practices. A low proportion of mothers in this study area demonstrated adequate knowledge and appropriate child‐feeding practices. Maternal education is associated with knowledge of complementary feeding. Similarly, maternal educational level, monthly average household income, and family size were identified as determinants of children's complementary feeding practices. Socioeconomic interventions, family planning programs, and nutritional education initiatives are essential to promote adherence to recommended feeding practices and caregiving approaches to enhance nutritional status and health outcomes in pediatric populations.

## Graphical abstract

Maternal knowledge and practices regarding complementary feeding in the Sedal District, Western Ethiopia, were found to be suboptimal, with only 46.8% of mothers demonstrating adequate knowledge and 42.7% practicing appropriate feeding. Maternal education, household income, and family size were significant determinants of these practices. Targeted socioeconomic interventions, family planning programs, and nutritional education initiatives are crucial to improving complementary feeding adherence and child health outcomes.

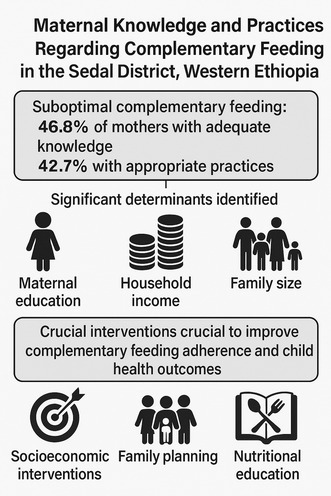

## 1 Introduction

Proper nutrition during early childhood is essential to ensure optimal growth, development, and cognitive performance (Abbas and Karim 2023; Black 2018). Adherence to recommended feeding practices and caregiving approaches is crucial for promoting healthy growth in children (Wood et al. 2020; World Health Organization 2004). Adequate nutritional intake not only supports immediate physical health but also significantly enhances future cognitive, motor, and social competencies (Tandon et al. 2016; Jirout et al. 2019). Conversely, inadequate nutrition may result in undernutrition and various health complications, potentially leading to adverse effects on cognitive function and overall well‐being (Kirolos et al. 2022; Precious et al. 2023).

The World Health Organization (WHO) advocates exclusive breastfeeding during the first 6 months of life, followed by the introduction of complementary foods while continuing breastfeeding for a minimum of 2 years (World Health Organization 2003). This approach is crucial for promoting the optimal growth and development of children. Complementary feeding refers to the introduction of solid foods in conjunction with breast milk to fulfill the nutritional requirements of infants after 6 months of age (Alvisi et al. 2015).

Adequate complementary feeding practices can help prevent malnutrition, which is a significant public health concern in many low‐income and middle‐income countries (White et al. 2017; Gatica‐Domínguez et al. 2021). Malnutrition during early childhood can lead to stunted growth, cognitive impairment, and increased susceptibility to infections (Acosta et al. 2018; Ibrahim et al. 2017). Complementary feeding should be timely, sufficient, safe, appropriate, and incorporate a variety of food groups to ensure a balanced diet (World Health Organization 2023; Harrison et al. 2023). Inadequate complementary feeding and improper caregiving practices during early childhood can lead to malnutrition and its associated health complications (Masuke et al. 2021; Bergamini et al. 2022). Low‐income countries, including Ethiopia, exhibit suboptimal complementary feeding practices that contribute to a high prevalence of micronutrient deficiencies in children (Gebretsadik et al. 2023; Ersino et al. 2016).

Maternal knowledge and practices regarding complementary feeding are critical determinants of enhanced child feeding and caregiving behaviors, which significantly influence infant nutrition and health outcomes (Arikpo et al. 2018; Herman et al. 2023). Maternal knowledge of complementary feeding encompasses an understanding of various food types and their nutritional contributions to the body, as well as their role in preventing malnutrition and enhancing health (Lutter et al. 2021; Keats et al. 2021). Complementary feeding practices also involve the introduction of appropriate solid foods and liquids alongside breast milk when an infant reaches 6 months of age (Caroli et al. 2021).

Mothers should possess adequate knowledge and implement appropriate practices regarding complementary feeding, including the timing of food introduction, continuity of breastfeeding alongside complementary foods, safety and adequacy of these foods, responsive feeding practices, food consistency, meal frequency, energy density, provision of a variety of nutrient‐rich foods, and the importance of good hygiene and proper food handling (Lutter et al. 2021; Afolabi et al. 2021; Dwijayanti et al. 2024; Ahmed et al. 2022b; Ajmal 2024; Manaseki‐Holland et al. 2021). In developing countries, infants and young children are particularly vulnerable to malnutrition owing to a lack of knowledge about proper feeding practices and inadequate caregiving approaches (Jibat et al. 2022; Forh et al. 2022). Maternal knowledge and practices related to complementary feeding may be influenced by various factors, such as sociocultural factors, socioeconomic status, traditional practices, poverty, and access to health care (Scarpa et al. 2022; Gatica‐Domínguez et al. 2021).

In Ethiopia, knowledge and practices concerning complementary feeding present significant challenges (Hirvonen et al. 2024; Assefa et al. 2021). The prevalence of inappropriate complementary feeding practices remains high, with variations observed across different regions. In central Ethiopia, more than half (55.3%) of the children demonstrated inadequate complementary feeding practices, which increased to 90.6% in agroecological areas (Gebretsadik et al. 2023; Gizaw et al. 2023). Additionally, approximately 60% of mothers lacked sufficient knowledge regarding complementary feeding (Abiyu and Belachew 2020).

Ethiopia is characterized by a rich diversity of ethnic groups, each with distinct cultural practices and dietary behaviors (Gebremichael and Belachew Lema 2023; Workneh et al. 2023). Rural and remote communities frequently encounter challenges associated with traditional customs and norms, exacerbated by limited access to healthcare communication, updated dietary guidelines, and modern services (Abdul et al. 2024; Shiferaw and Zolfo 2012). Sedal District exemplifies such a rural area, which is located at a considerable distance from the country's urban centers. Local conflicts may adversely affect dietary practices by disrupting food production.

Assessing maternal knowledge and practices related to complementary feeding in the Sedal District is essential for understanding and improving child nutrition. This study aimed to evaluate maternal knowledge and practices regarding complementary feeding in Ethiopia. The findings of this study could provide nutritional recommendations for children within the community. Furthermore, the results will provide valuable insights for health and nutrition authorities, as well as other stakeholders, to enhance child‐feeding practices throughout the country.

## 2 Methods and Materials

### 2.1 Study Design, Population, and Setting

A community‐based cross‐sectional study was conducted among mothers of children aged 6–23 months in Sedal District from February to July 2023. This district is situated in the Kemish Zone of the Benishangul Gumuz region, approximately 654 km west of Addis Ababa, Ethiopia's capital. The district has a population of 27,857, including 14,056 men and 13,801 women. According to local data, there were 2864 children aged 6–23 months in the district. The study area consisted of 14 rural kebeles and one urban center.

### 2.2 Eligibility Criteria

Mothers of children aged 6–23 months who had resided for more than 6 months in randomly selected kebeles within the cluster and were willing to participate were included in the study. Mothers whose children reported mental or chronic illnesses based on their self‐reports were excluded from the study.

### 2.3 Sample Size Determination and Sampling Techniques

The required sample size for this study was calculated using a single‐population proportion formula based on a previous finding of 49%, with a 95% confidence level and 5% margin of error (Berisha et al. 2017). After accounting for a nonresponse rate of 10%, the final sample size was 422 participants. This study used the cluster and random sampling methods. Initially, the district was divided into four distinct clusters based on its geographical location, with one kebele randomly selected for each cluster. Subsequently, using local government reports on the number of children in the selected kebeles, the sample size was proportionally allocated, and interviews with mothers were conducted. House‐to‐house visits were conducted to determine the target number of women. After selecting an eligible child, the mother or primary caregiver was interviewed.

### 2.4 Operational Definitions

#### 2.4.1 Maternal Knowledge of Complementary Feeding

Knowledge of complementary feeding pertains to mothers' understanding of the introduction of semi‐solid or solid foods to infants in conjunction with breastfeeding, particularly when breast milk alone no longer meets the nutritional requirements of the child.

Knowledgeable: If respondents score prepared knowledge questions ≥ mean.

Not knowledgeable: If respondents score prepared knowledge questions < mean.

#### 2.4.2 Maternal Practices Regarding Complementary Feeding for Children

Complementary feeding practices refer to the introduction of solid foods and liquids to an infant's diet alongside breast milk, typically starting at around 6 months of age.

Appropriate: If respondents score ≥ mean prepared questions of their child's feeding practices.

Inappropriate: If respondents score < mean prepared questions of their child's feeding practices.

### 2.5 Data Collection Procedures

Data were collected using a semi‐structured questionnaire adapted and modified based on the literature (Gebretsadik et al. 2023; Dwijayanti et al. 2024; Ahmed et al. 2022b; Berisha et al. 2017). The questionnaire was initially developed in English, translated into the local language, and subsequently back‐translated into English by proficient speakers of both languages to ensure accuracy. The questionnaire included inquiries related to sociodemographic characteristics, obstetric and health‐related factors, and mothers' knowledge and practices concerning complementary feeding for children aged 6–23 months. Mothers who scored at or above the mean score on the prepared knowledge questions on complementary feeding were classified as knowledgeable. Similarly, mothers who scored at or above the mean score on the prepared questions concerning their children's complementary feeding practices were deemed to have appropriate complementary feeding practices.

To enhance the data collection process, three health extension workers from each of the selected kebeles and nine data collectors were trained. The principal supervisor conducted training sessions that focused on the study's objectives, purpose, and interviewing techniques in accordance with the research instrument. Furthermore, the training included instructions on questionnaire administration and probing strategies to obtain detailed responses.

### 2.6 Data Quality Control

All completed questionnaires were thoroughly examined for incomplete data entry. Throughout the data entry process, verification checks were conducted at regular intervals to identify and rectify inconsistencies. Data cleaning was performed prior to analysis.

### 2.7 Statistical Analysis

The cleaned and coded data were entered into EpiData 3.1 software and subsequently exported to IBM SPSS version 25 for analysis. Maternal sociodemographic characteristics, along with awareness and practice, were summarized using descriptive statistics. Bivariate and multivariable logistic regression analyses were performed to investigate the association between the dependent and independent variables. Variables with a *p* value of less than 0.2 in the bivariate regression analysis were included in the multivariable logistic regression analysis, with a *p* value of less than 0.05 deemed statistically significant. Prior to the final logistic regression model analysis, we assessed assumptions related to multicollinearity and confounding factors. The fit of the final model was evaluated using the Hosmer–Lemeshow goodness‐of‐fit test.

## 3 Results

### 3.1 Sociodemographic and Economic Characteristics of the Study Participants

In the present study, the response rate was 97.2%. Only 5.4% of the study participants had a college education or higher. The majority (96.8%) of mothers were married, and 92.2% of mothers were housewives by occupation. Among the husbands, 80.7% were farmers (Table 1).

**TABLE 1:** Sociodemographic and economic characteristics of participants in the Sedal district in western Ethiopia.

Variable	Frequency (*n*)	Percent
Mothers age		
< 20	172	42.0
21–25	132	32.2
26–30	87	21.2
31–35	12	2.9
> 35	7	1.7
Mothers' marital status		
Married	397	96.8
Others^a^	13	3.2
Mothers' education		
No formal education	161	39.3
Primary and secondary school	227	55.4
College and above	12	2.9
Husband education		
No formal education	152	37.1
Primary and secondary schools	240	58.5
College and above	18	4.4
Mothers' occupation		
Housewife	374	91.2
Government employee	24	5.9
Others^b^	12	2.9
Husband occupation		
Farmer	331	80.7
Government employee	46	11.2
Private	33	8.1
Monthly average income		
< 1500	225	54.9
1500–3000	163	39.7
> 3000	22	5.4
Household size		
1–3	141	34.4
3–6	250	61.0
> 6	19	4.6
Childs age		
6–11	78	19.1
12–17	174	42.4
18–23	158	38.5
Childs sex		
Male	208	50.7
Female	202	49.3
Childs birth order		
First	20	4.9
2–3	122	29.8
> 3	268	66.1

^a^ Divorced, widowed.

^b^ Farmer, Self employed, Merchant.

### 3.2 Maternal Knowledge on Complementary Feeding

Overall, 46.8% of the mothers were knowledgeable about complementary feeding. Regarding the specific knowledge proportions, 26.5% of the mothers understood the correct definition of complementary feeding. Approximately half (50.2%) of the mothers knew the appropriate time for the initiation of complementary foods, while 76.1% knew the frequency and content of these foods. Additionally, 60.2% of the mothers were aware of the importance of utensils and hygiene during complementary feeding. The majority of mothers understood the risks associated with the late initiation of complementary foods (Table 2).

**TABLE 2:** Maternal knowledge on complementary feeding in Sedal district, western Ethiopia.

Variables (questions)	Knew (*n*)	Percent
Definition of complementary feedings	109	26.5
Time initiation of complementary foods	206	50.2
Minimum years for BF with continued CF	79	19.3
Preparation of complementary foods	165	40.2
Risk of late initiation of complementary foods	370	90.2
Complementary feeding utensils and hygiene	247	60.2
Frequency and content of complementary foods	312	76.1
Overall knowledge		
Knowledgeable	192	46.8
Not knowledgeable	218	53.2

### 3.3 Maternal Practices on Child's Complementary Feeding

In this study, the overall prevalence of appropriate complementary feeding among children aged 6–23 months was 42.7%. Regarding the specific knowledge proportion, timely initiation of complementary feeding accounted for 42.3%. The minimum meal frequency and dietary diversity were 45.6% and 49.3%, respectively. Approximately 43.7% of the participants practiced safe hygiene using utensils (Tables 3 and 4).

**TABLE 3:** Child complementary feeding practices in Sedal district, western Ethiopia.

Variables	Frequency	Percent
Timely introduction of complementary feeding		
Yes	174	42.3
No	236	57.7
Minimum meal frequency		
Yes	187	45.6
No	223	54.4
Minimum dietary diversity		
Yes	202	49.3
No	208	49.6
Child‐feeding utensils and hygiene		
Yes	179	43.7
No	231	56.3
Currently breast feeding		
Yes	393	95.8
No	17	4.2
Overall complementary feeding practices		
Appropriate	175	42.7
Inappropriate	235	57.3

**TABLE 4:** Factors associated with maternal knowledge and practices in Sedal district, western Ethiopia.

Variables	Knowledge		AOR	Practices		AOR
	Knowledgeable	Not knowledgeable	95% CI	Appropriate	Inappropriate	95% CI
Mothers' education						
No formal education	82	79	1	65	96	1
Primary and secondary school	94	133	1.1 (0.88–3.03)	96	131	1.9 (0.57–5.39)
College and above	16	6	2.3 (1.06–5.10)^a^	14	8	2.4 (1.48–4.01)^a^
Husband education						
No formal education	72	80	1	69	83	1
Primary and secondary school	101	139	0.7 (0.12–0.06)	107	133	1.2 (0.75–1.72)
College and above	11	9	1.32 (0.82–2.11)	7	13	1.6 (0.85–2.94)
Husband occupation						
Farmer	141	190	1	137	194	1
Government employee	27	19	1.4 (0.47–3.86)	24	22	0.39 (0.10–1.51)
Private	17	16	1.3 (0.28–3.82)	19	14	1.52 (0.59–3.93)
Monthly average income (ETB)						
< 1500	111	114	1	108	117	1
1500–3000	74	89	0.39 (0.19–0.79)	71	92	1.02 (0.15, 6.75)
> 3000	16	6	1.30 (0.78–2.15)	15	7	2.7 (1.54–4.57)^a^
Household size						
1–3	88	53	1	85	56	1
3–6	113	137	1.6 (0.85–2.94)	110	140	1.76 (1.00–3.09)
> 6	8	11	1.4 (0.47–3.86)	9	10	3.952 (2.12–7.36)^a^
Childs birth order						
First	9	11	1	8	12	1
2–3	49	73	1.32 (0.73–2.41)	51	71	1.9 (0.57–5.39)
> 3	131	137	1.18 (0.35–3.94)	126	142	1.7 (0.88–3.56)

Abbreviations: 1, Reference; AOR, adjusted odds ratio; ETB, Ethiopian Birr.

^a^ Significant variables.

### 3.4 Factors Associated With Maternal Knowledge and Practices on Complementary Feeding

In bivariate logistic regression analysis, mothers' education, husbands' education, and household monthly average income were predicted to be associated with mothers' knowledge of commentary feeding. However, in the multivariable analysis, only the mother's education was significantly associated with maternal knowledge of complementary feeding. Similarly, in bivariate logistic regression analysis, mothers' education, husbands' education, husbands' occupation, household monthly average income, household size, and birth order were predicted to be associated with mothers' feeding practices. However, in the multivariate analysis, determinant factors such as mothers' education, household monthly average income, and household size were significantly associated with mothers' feeding practices in the study area.

## 4 Discussion

Adhering to recommended feeding practices and caregiving approaches is vital for fostering healthy growth in children. Inadequate complementary feeding and improper caregiving practices during early childhood can lead to malnutrition and associated health complications. Low‐income countries, including Ethiopia, exhibit suboptimal complementary feeding practices that contribute to the high prevalence of micronutrient deficiencies in children. Insufficient knowledge and inappropriate practices are challenges associated with multiple dimensions. It is important to assess maternal knowledge and practices of complementary child feeding to improve child nutrition, particularly for children who are far from centers in the country. Therefore, this study assessed maternal knowledge and practices regarding the complementary feeding of children in the Sedal District of Western Ethiopia. Overall, 46.8% and 42.7% of the mothers were knowledgeable and had appropriate practices regarding their children's complementary feeding, respectively. Women's educational level was significantly associated with maternal knowledge of complementary feeding. Likewise, Women's education, household monthly average income, and family size were significantly associated with mothers' children's complementary feeding practices.

In this study, 46.8% of mothers demonstrated familiarity with complementary feeding, which is notably lower than the percentages observed in similar studies conducted in Northwest Ethiopia (60%), Ghana (52%), and Southwest Ethiopia (52.5%) (Gizaw et al. 2023; Abiyu and Belachew 2020; Bimpong et al. 2020). Conversely, this figure is higher than the findings from other studies in Ethiopia, where only 9.76% and 21.71% of mothers exhibited knowledge regarding complementary feeding practices for their children, respectively (Shagaro et al. 2021; Dagne et al. 2022). The discrepancies observed can be confidently attributed to several key factors, including sociodemographic variations within communities and differences in health service utilization. These elements crucially affect the accessibility of information regarding children's feeding practices.

The findings of this study indicate that an increase in women's educational level is significantly associated with enhanced maternal knowledge of complementary feeding. This correlation suggests that education plays a crucial role in fostering a better understanding of proper child‐feeding practices. Educated mothers were more likely to comprehend nutritional and health messages from various sources. These results align with similar studies conducted in Southwest Ethiopia, Bangladesh, and Nigeria, reinforcing the importance of education in promoting effective child nutritional practices (Tadegew et al. 2024; Owais et al. 2019; Olatona et al. 2017).

In this study, 42.7% of the mothers demonstrated appropriate practices concerning their children's complementary feeding. This finding aligns with those of other studies conducted in Ethiopia, which reported that 46.7% and 45.3% of women practiced adequate complementary feeding for their children (Feleke et al. 2021). However, our results are lower than those observed in Shashemene, Southern Ethiopia (55.3%), and Gurage Zone, Central Ethiopia (54.4%) (Kasse et al. 2024; Ayu et al. 2024). Notably, our findings surpass those of a study in Tigray, Ethiopia, where only 19% of mothers were found to have adequate child‐feeding practices (Mulugeta et al. 2024).

The observed differences may be attributed to sociodemographic and economic variations, cultural norms, and the availability of food resources. Various regions of Ethiopia continue to experience conflict, presenting public challenges that can significantly affect food availability and living standards within communities. The study area has faced different types of conflicts, which may influence both the social and economic dimensions of life for residents.

The current study revealed a significant correlation between the level of education attained by mothers and complementary feeding practices for their children. Specifically, mothers with a higher educational background exhibited a more comprehensive understanding and effective implementation of appropriate feeding strategies during the critical complementary feeding period, which plays a vital role in a child's growth and development. These findings align with previous research conducted in Southern Ethiopia, Ghana, and Nigeria, reinforcing the importance of maternal education in promoting optimal nutritional practices for children (Kasse et al. 2024; Issaka et al. 2015; Udoh and Amodu 2016).

Monthly average household income significantly influenced maternal complementary feeding practices for children. Families with higher income levels typically have better access to a diverse range of nutritious foods, enabling mothers to offer more balanced and varied diets to their children. In contrast, lower‐income households often encounter challenges such as food insecurity, which can restrict the availability of healthy food options and result in less optimal feeding practices. Over the past 6 years, various communities in Ethiopia have faced economic inflation and political instability, exacerbating these challenges. This study draws parallels with similar research conducted in Pakistan, Indonesia, Ethiopia, and Nepal (Ali et al. 2021; Nurrizka et al. 2021; Ahmed et al. 2022a; Gurung et al. 2024).

This study highlighted a notable correlation between family size and complementary feeding practices adopted by mothers for their children. The number of siblings a child has can significantly affect the timing and manner in which complementary foods are introduced as well as the diversity and nutritional quality of these foods. In larger families, mothers often encounter greater challenges in distributing time and resources to their children, which can adversely affect their capacity to offer varied and nutritious complementary food options. This study aligns with findings from similar studies conducted in Tanzania, Kenya, and Western Ethiopia (Masuke et al. 2021; Uusimäki et al. 2023; Jebena and Tenagashaw 2022).

### 4.1 Strength and Implication of the Study

The strength of this conclusion lies in its comprehensive analysis of the factors affecting maternal knowledge and practices of complementary feeding in Western Ethiopia's Sedal District. It highlights key correlations between maternal education, household income, family size, and feeding practices, emphasizing the importance of education in promoting proper child nutrition. The findings also consider regional differences and socioeconomic challenges, providing a context for observed variations. By linking these factors to relevant literature, this study underscores the need for targeted interventions, such as improving maternal education and addressing socioeconomic barriers, to enhance feeding practices and reduce child malnutrition.

### 4.2 Limitations

The study gathered information from mothers about their past experiences, which could have introduced recall bias. Furthermore, the cross‐sectional design makes it challenging to establish cause‐and‐effect relationships.

## 5 Conclusion

In conclusion, this study highlights the critical role of maternal knowledge and effective practices in complementary feeding to enhance child nutrition and mitigate malnutrition, particularly in resource‐constrained environments such as Ethiopia. The findings indicated a notable prevalence of maternal awareness and practices, with only 46.8% of mothers displaying sufficient knowledge and 42.7% achieving appropriate practices. The key determinants of maternal practices in complementary feeding include women's education, household income, and family size.

These findings underscore the importance of education in improving mothers' comprehension and execution of effective feeding strategies. Additionally, they highlight the need to address socioeconomic barriers that restrict access to various nutritious foods. These insights advocate focused interventions that encompass educational initiatives and policies designed to enhance maternal knowledge and improve economic circumstances. Such efforts are crucial for fostering optimal child‐feeding practices. By addressing these gaps, we can make significant strides in reducing malnutrition and promoting healthy growth and development of children in Ethiopia and comparable settings.

## Author Contributions

**Habtamu Fekadu Gemede:** conceptualization (equal), data curation (equal), formal analysis (equal), methodology (equal), software (equal), supervision (equal), writing – original draft (equal), writing – review and editing (equal). **Kassahun Ayele:** conceptualization (equal), data curation (equal), formal analysis (equal), methodology (equal), software (equal), supervision (equal), writing – original draft (equal), writing – review and editing (equal). **Meron Demisew:** conceptualization (equal), data curation (equal), formal analysis (equal), methodology (equal), software (equal), supervision (equal), writing – original draft (equal), writing – review and editing (equal).

## Ethics Statement

This study was conducted in accordance with the guidelines set forth in the Declaration of Helsinki and approved by the ethics review committee of Wollega University.

## Consent

Informed consent was obtained from all participants prior to their inclusion in the study.

## Data Availability

The datasets used and/or analyzed during the current study are available from the corresponding author upon reasonable request.
